# Thermomechanical Response of Fullerene-Reinforced Polymers by Coupling MD and FEM

**DOI:** 10.3390/ma13184132

**Published:** 2020-09-17

**Authors:** Georgios I. Giannopoulos, Stelios K. Georgantzinos, Nick K. Anifantis

**Affiliations:** 1 Department of Mechanical Engineering and Aeronautics, School of Engineering, University of Patras, GR-26500 Patras, Greece; nanifantis@upatras.gr; 2 Laboratory for Advanced Structures and Smart Systems, General Department, National and Kapodistrian University of Athens, 15772 Psachna, Greece; sgeor@uoa.gr

**Keywords:** nanocomposite, PMMA, fullerene, finite element method, molecular dynamics, multiscale

## Abstract

The aim of the present study is to provide a computationally efficient and reliable hybrid numerical formulation capable of characterizing the thermomechanical behavior of nanocomposites, which is based on the combination of molecular dynamics (MD) and the finite element method (FEM). A polymeric material is selected as the matrix—specifically, the poly(methyl methacrylate) (PMMA) commonly known as Plexiglas due to its expanded applications. On the other hand, the fullerene C_240_ is adopted as a reinforcement because of its high symmetry and suitable size. The numerical approach is performed at two scales. First, an analysis is conducted at the nanoscale by utilizing an appropriate nanocomposite unit cell containing the C_240_ at a high mass fraction. A MD-only method is applied to accurately capture all the internal interfacial effects and accordingly its thermoelastic response. Then, a micromechanical, temperature-dependent finite element analysis takes place using a representative volume element (RVE), which incorporates the first-stage MD output, to study nanocomposites with small mass fractions, whose atomistic-only simulation would require a substantial computational effort. To demonstrate the effectiveness of the proposed scheme, numerous numerical results are presented while the investigation is performed in a temperature range that includes the PMMA glass transition temperature, *T*_g_.

## 1. Introduction

Commonly, the nanocomposite materials applications are associated with the simultaneous action of more than one type of loading. Especially, the investigation of nanocomposites subjected to both thermal as well as mechanical loads is perhaps one of most interesting fields for research, since high-temperature applications are very frequent. Recently, polymers that are reinforced with carbon nanomaterials have greatly attracted the scientific interest because of their enhanced material properties such as high strength-to-weight ratio. Evidently, the characterization of the thermomechanical performance of such nanocomposites may offer versatile design solutions for a variety of novel applications. In an effort to highlight significant innovations and potential applications in this research area, Burgaz [1] has investigated the current status of thermomechanical properties of polymers containing nanofillers in the form of nanocylinders, nanospheres, and nanoplatelets.

Since the experimental procedures intended for an adequate characterization of nanostructured composites are complicated and require extensive resources and time, the development and introduction of new computational approaches for simulating nanocomposites may be considered as a valuable, if not necessary, alternative. Perhaps, the MD method is the most popular tool for analyzing nanomaterial-reinforced polymers due to its ability to capture, with high accuracy, all the interatomic phenomena with respect to the temperature and pressure.

Discussion on some of the most interesting MD studies associated with the thermomechanical response of nanoreinforced polymers is essential. In a relatively early study, Cho and Yang [2] have performed a parametric study to investigate the effects of composition variables on the thermal and mechanical properties of carbon nanotube (CNT) reinforced polymers using MD simulations. Given that the glass transition temperature *T*_g_ is a key property for polymers, Allaoui and Bounia [3] have reviewed and analyzed various literature results dealing with the effect of unmodified multiwall carbon nanotubes (MWCNT) on the cure kinetics and *T*_g_ of their epoxy composites. Aiming at a similar goal, Herasati et al. [4] have investigated the effects of polymer chain branches, crystallinity, and CNT additives on the glass transition temperature of polyethylene (PE). Targeting a different matrix material, Mohammadi et al. [5] have investigated the effect of alumina and modified alumina nanoparticles on the glass transition behavior of a PMMA/alumina nanocomposite by MD simulations. The effect of inorganic particles have been studied by Zhang et al. [6], who have established silica–epoxy nanocomposite models to investigate the influence of a silane-coupling agent on the structure and thermomechanical properties of the nanocomposites through MD simulation. An extended study has been performed by Pandey et al. [7] focusing on the computation of viscoelastic, thermal, electrical, and mechanical properties of graphite flake-reinforced high-density PE composites. Recently, Dikshit and Engle [8] have employed MD simulations to study the mechanical properties of epoxy bisphenol A diglycidyl ethe (DGEBA) with and without the reinforcement of CNT, while in a similar attempt, Dikshit et al. [9] have performed a MD study to investigate the mechanical properties of graphene-reinforced epoxy nanocomposite. Aiming on the study of functionalizing polymer carbon nanofillers, Xue [10] have performed a cooling process by MD simulation to predict the *T*_g_ of graphene/PMMA composites. On the other hand, for the first time, Park et al. [11] investigated the thermomechanical characteristics of silica-mineralized nitrogen-doped CNT-reinforced PMMA nanocomposites by MD simulations. An interesting investigation regarding the interfacial behavior of functionalized CNT/PE nanocomposites at different temperatures has been performed by Singh and Kumar [12] using MD simulations and the second-generation polymer consistent force field (PCFF). Experimenting in a differently nanostructured reinforcing agent, Zhang et al. [13] have investigated via MD simulations the thermomechanical properties of nanocomposites consisting of weaved PE and CNT junctions.

Although there have been numerous efforts to investigate the influence of dispersing CNTs and graphene nanoribbons in polymers, fewer studies are available on the relevant effects of spherical carbon nanoparticles. In a study distinguished because of the kind of carbon allotrope that is used as a nanoreinforcement, Jeyranpour et al. [14] have adopted MD to carry out a comparative study regarding the effects of fullerenes on the thermomechanical properties of a specialized resin epoxy. Izadi et al. considered a similar nanocomposite [15] when estimating the elastic properties of PMMA reinforced with C_60_ fullerene and C_60_@C_240_ carbon onion by using MD simulations; however, they did not consider the effect of temperature.

All the above investigations have been realized via MD, which is a method that demands extensive computational power. Due to the high pre-processing and main-processing computational times required for analyzing material components at the nanoscale, several multiscale techniques [16,17,18] have been proposed that combine the benefits of molecular and continuum modeling. Characteristically, Montazeria and Rafii-Tabar [16] presented a combination of MD, molecular mechanics (MM), and the finite element method (FEM) that is capable of computing the elastic constants of a polymeric nanocomposite embedded with graphene sheets and carbon nanotubes at various temperatures. Similarly, Tsiamaki and Anifantis [17] have utilized a multiscale model based on MM and FEM to analyze the thermomechanical behavior of graphene/PMMA nanocomposites. Recently, Giannopoulos [18] proposed a formulation combining MD and FEM to predict the mechanical behavior of fullerene-reinforced nylon-12; however, this was at room temperature only. An interesting review on the recent developments in multiscale modeling of the thermal and mechanical properties of advanced nanocomposite systems has been given by Reddy et al. [19].

Apart from the more common carbon nanomaterials such as CNTs and graphene, which have unquestionably attracted the most attention in recent years, many researchers have started to explore the effects of reinforcing polymers with fullerenes [14,15,18]. Especially giant fullerenes such as C_240_ present unique characteristics that have been widely studied in the recent years. Giant fullerenes have already been experimentally observed and successfully produced. Very early, Ruoff et al. [20] utilized mass-spectrometric techniques to demonstrate the presence of carbon clusters C_2*n*_ with *n* as high as 300, in carbon soot material produced using the arc-synthesis method. On the other hand, Shinohara et al. [21] successfully extracted a series of very large all-carbon molecules, including C_240_, with quinoline from fullerene-rich carbon soot produced by the vaporization of graphite in a helium atmosphere using the contact arc method. In an effort to provide generalized geometrical relationships describing the structure of giant fullerenes, Wang and Chiu [22] have also shown that the C_240_ giant fullerene cage has the same I_h_ symmetry as C_60_ and that it has twelve pentagonal faces in icosahedra alignment. Having a similar aim, Schwerdtfeger et al. [23] recently presented a general overview of recent topological and graph theoretical developments in fullerene research over the past two decades, describing both solved and open problems. In the theoretical field, Kim and Tomnek [24] reported an MD simulation of melting and evaporation of the carbon fullerenes C_20_, C_60_, and C_240_. Finally, focusing on the C_240_, Cabrera-Trujillo [25] used density functional theory (DFT) to study the electronic structure and binding of Na clusters encapsulated inside the fullerene cage.

Considering the exceptional structural and physical properties of giant fullerenes, which have been extensively discussed in some of the aforementioned studies, the reinforcing capability of C_240_ when compounded with polymers is computationally investigated in the present study over a wide temperature range. The symmetric fullerene C_240_ is preferred as a reinforcing agent because of its high symmetry, which may allow the achievement of an almost isotropic nanocomposite behavior. In addition, the PMMA is selected as the matrix material due to its high stiffness and wide range of applications. Moreover, its sensitivity on the temperature around its glass transition point, which has been investigated in several experimental [26,27,28] and MD studies [29], may permit the drawing of more illustrative conclusions about the fullerene reinforcement impact under different loading and environmental conditions. The adopted numerical technique is performed at two scales. At the first scale, MD simulations [30] of a low computational cost are performed by using a periodic unit cell to extract the temperature-dependent properties of the pure PMMA as well as the C_240_/PMMA nanocomposite at a high mass fraction of 20%. The Condensed Phase Optimized Molecular Potential (COMPASS) [31] is adopted in view of its superiority over other potential models describing polymers [32]. Then, at a second scale, an RVE is developed and simulated via FEM [33] by using the data outputs from the MD-only analysis, in order to investigate nanocomposites with small C_240_ mass fractions, whose analysis via MD would not be computationally feasible by utilizing typical computer resources. A variety of diagrams are presented that depict the variation of nanocomposite properties such as elastic modulus, Poisson’s ratio, and linear coefficient of thermal expansion with temperature and C_240_ mass fraction. Comparisons with relevant predictions found elsewhere are attempted, where possible. To the author’s best knowledge, it is the first time that the temperature-dependent mechanical properties of the C_240_/PMMA nanocomposite are predicted via a multiscale technique based on MD and FEM.

## 2. Multiscale Analysis

It is well known that MD is a numerical simulation method that is capable of predicting the time evolution of a system of interacting atoms. It is based on the generation of atomic trajectories via the numerical integration of Newtown’s equation of motion by utilizing a specific interatomic potential, initial conditions, and boundary conditions. Although the MD method may accurately represent all the interatomic phenomena, it entails a substantial computational cost, which is dramatically increased with the number of the interacting atoms [18], due to the numerical integrations over long time intervals that are usually required to reach equilibrium states. Thus, the analysis of large systems such as the one tested here, i.e., a C_240_/PMMA nanocomposite, leads to the necessity of combining atomistic with continuum numerical approaches. The use of a multiscale technique becomes a must when dealing with composites reinforced with low mass fractions of nanoparticles, since their MD-only analysis would require extremely large periodic unit cells.

**Figure 1:**
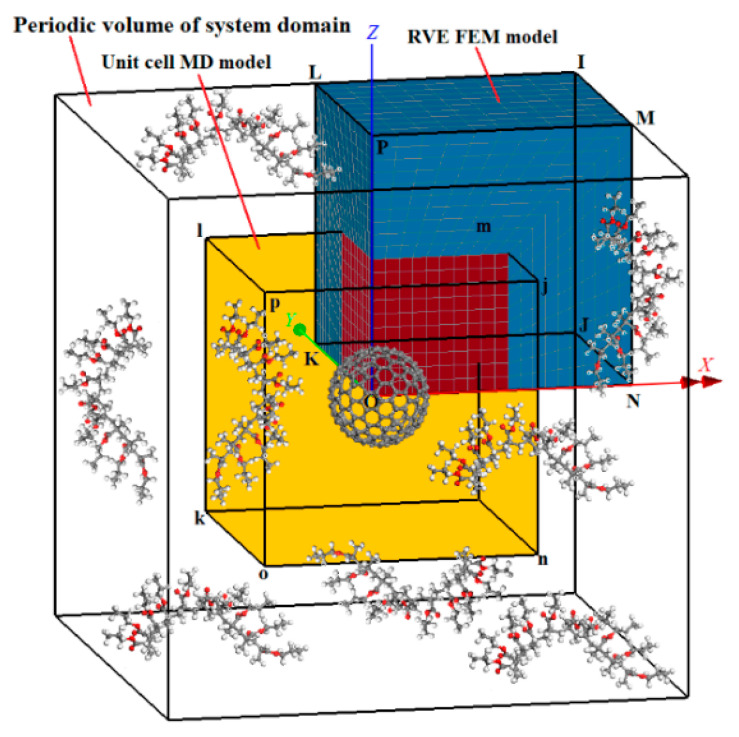
The nanocomposite representative volume element (RVE) (1/8 of the periodic volume of the system domain) with a small fullerene mass fraction and the nanocomposite unit cell with a fullerene mass fraction of 0.2, modeled via the finite element method (FEM) and molecular dynamics (MD), respectively.

Let us assume that the investigated C_240_/PMMA nanocomposite is characterized by a uniform and periodic reinforcement distribution. Given the spherical and symmetric shape of C_240_ nanoparticles, the system domain may be fully described by a cubic periodic volume of the system domain illustrated in Figure 1, which contains a centrally located fullerene surrounded by a number of PMMA chains, i.e., the matrix material.

The adopted numerical analysis is contacted at two scales. The MD-only method is utilized at the first scale while a CM method, realized via FEM and by using the previous MD output data, is performed at the second scale. The MD method offers the important precise representation of interfacial interactions and stress transfer mechanisms between the fullerene and the matrix while the CM method, based on FEM, provides modeling simplicity and a low computational cost.

At the first scale, MD simulations of the pure PMMA with respect to the temperature are initially performed to extract the necessary temperature-dependent property curves for the matrix material. Then, another periodic unit cell is developed and simulated that represents a nanocomposite with a high weight concentration of C_240_ equal to *w*_C240_ = 0.2. The MD-only simulation of the specific nanocomposite unit cell, whose topology is defined by the yellow colored ijklmnop cubic domain in Figure 1, leads to the computation of corresponding temperature-dependent property curves.

At the second scale, a CM-based RVE, denoted as ijklmnop in Figure 1, is developed for three small mass fractions of C_240_, i.e., *w*_C240_ = 0.01, 0.03, and 0.05, and then, it is simulated through FEM using appropriate boundary conditions of symmetry and loading. Note that only the 1/8 of the periodic volume of the whole system domain is required to be represented due to the symmetry of the nanoparticle and its assumed uniform distribution within PMMA. Evidently, the red-colored subdomain of the RVE close to the vertex O (common volume between ijklmnop and IJKLMNOP cubes) is governed by the thermomechanical behavior of the nanocomposite with *w*_C240_ = 0.2, while the remaining blue-colored subdomain of the RVE represents the pure PMMA matrix. The temperature-dependent material properties, extracted from the MD-only simulations at the first scale analysis, are utilized in order to enable the finite element simulation of both RVE subdomains. The material properties in both subdomains are considered isotropic elastic, given that very small static strains are applied for the requirement of the present study.

## 3. First Simulation Scale: MD-Only Formulation

### 3.1. Unit Cells Construction

**Figure 2:**
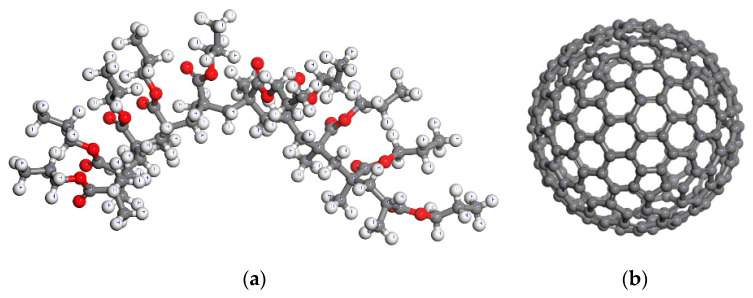
Molecular structure of the: (**a**) poly(methyl methacrylate) (PMMA) chain and (**b**) C_240_ fullerene.

At the first scale of the analysis, as aforementioned, two different simulation stages take place. Initially, the pure PMMA is analyzed at various temperatures by utilizing a large enough unit cell to ensure convergence. Evidently, as the pure PMMA unit cell becomes larger, the number of polymer chains increases. Furthermore, when the simulation box contains a high number of polymer chains, its response becomes statistically independent of the relative chain nanostructural positioning and alignment. As a result, the MD-based numerical solution remains stable for large unit cells. Here, a series of polymeric chains of 10 monomers each are adopted in order to represent the PMMA, as Figure 2a depicts.

**Figure 3:**
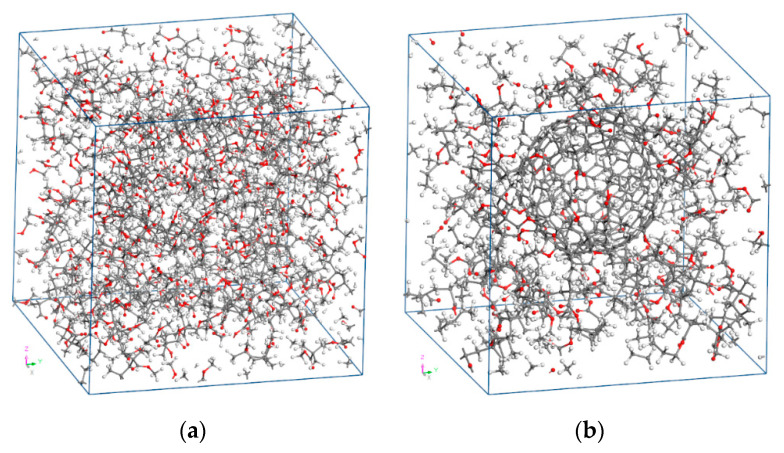
Equilibrated unit cell at the temperature *T* = 300 K of the (**a**) pure PMMA and (**b**) fullerene-reinforced PMMA at a mass fraction of *w*_C240_ = 0.2.

The pure PMMA unit cell is analyzed according to a global Cartesian coordinate system (*x*, *y*, *z*) for each tested temperature level. When performing MD simulations, it is very convenient to initially adopt a small unit cell density in order to obtain a sparse molecular distribution. Then, common equilibrium algorithms are applied at each time point, to obtain the actual density of the unit cell as well as its equilibrated configuration. Here, it is assumed that the PMMA has an initial density at the room temperature *T* = 300 K equal to 0.6 g/cm^3^. According to this PMMA density value, by utilizing 20 polymer chains and by taking into account the molecular weight of each chain, a cubic periodic unit cell may be defined [30]. It should be noted that the adoption of more than 20 chains inside the unit cell has been shown to have a negligible effect on the computed thermomechanical response of the pure PMMA. After conducting the full MD procedure described in the following section, the converged variations of volume, density, elastic modulus, and Poisson’s ratio with the temperature are obtained. The equilibrated amorphous unit cell of the pure PMMA at 300 K is illustrated in Figure 3a.

Secondly, the initial structure of the nanocomposite unit cell with *w*_C240_ = 0.2 is defined in a more complicated manner. First of all, the fullerene C_240_ of Figure 2b is maintained at the center of the unit cell at all times. The average radius of the specific fullerene is about *r*_C240_ = 7.07 Å [22,23]. In addition, its wall thickness is assumed to be equal to the usual distance between two successive carbon layers in graphite, i.e., *t* = 3.35 Å. Given, the specific wall thickness and the almost spherical shape of the tested fullerene, its density at the room temperature may be approximated by the following equation:

(1) $$\rho_{C240}=\frac{4}{3}\frac{240m_{C}}{\pi {\left(r_{C240}+t/2\right)}^{3}}$$

where *m*_C_ = 1.9927 × 10^−23^ g is the mass of a carbon atom.

In order to enable packing [30] of the PMMA chains into the unit cell, an initial nanocomposite density of 0.6 g/cm^3^ is beforehand assumed for the room temperature. Then, the initial nanocomposite unit cell volume may be estimated by the following relationship:

(2) $$V=\frac{m_{C240}}{w_{C240}\rho_{C240}}$$

Finally, before the initial packing of PMMA chains inside the unit cell and around the central positioned fullerene, the following nanocomposite unit cell length may be assumed:

(3) $$L=\sqrt[{3}]{V}$$

After having defined the size of the three-dimensional (3d) nanocomposite unit cell for the room temperature, a number of PMMA chains are inserted into it, while the packing algorithm evenly increases their population until the initial assumed density is achieved. The equilibrated unit cell of the nanocomposite with *w*_C240_ = 0.2 at 300 K is shown in Figure 3b.

### 3.2. Geometry Optimization of Molecular Structures and Potential Model

Firstly, geometric optimization (GO) [30] is performed for each initially assumed molecular structure, i.e., the main PMMA chain as well as the C_240_ fullerene, which are depicted in Figure 2a,b, respectively. During the GO, energy minimization is achieved by using the steepest descent algorithm [30]. It is assumed that convergence is accomplished when the absolute difference of the computed system energy and force between two subsequent iterations becomes less than 0.001 Kcal/mol and 0.5 Kcal/mol/Å, respectively. The required numerical calculations are based on the COMPASS potential, which consists of the ten valence terms and two non-bonded interaction terms given below [31].

(4) $$\begin{array}{l} U=\sum_{\mathrm{bond}}[k_{b2}{\left(b-b_{0}\right)}^{2}+k_{b3}{\left(b-b_{0}\right)}^{3}+k_{b4}{\left(b-b_{0}\right)}^{4}]+\sum_{\mathrm{angle}}[k_{a2}{\left(\theta -\theta_{0}\right)}^{2}+k_{a3}{\left(\theta -\theta_{0}\right)}^{3}+k_{a4}{\left(\theta -\theta_{0}\right)}^{4}]\\ +\sum_{\mathrm{torsion}}\left[k_{t1}(1-\cos\phi \right)+k_{t2}(1-\cos2\phi )+k_{t3}(1-\cos3\phi )]+\sum_{\mathrm{out}\;\mathrm{of}\;\mathrm{plane}\;\mathrm{angle}}k_{\chi }{\left(\chi -\chi_{0}\right)}^{2}+\sum_{\mathrm{bond}/\mathrm{bond}}k_{bb}(b-b_{0})(b^{\prime }-{b^{\prime }}_{0})\\ +\sum_{\mathrm{bond}/\mathrm{angle}}k_{ba}(b-b_{0})(\theta -\theta_{0})+\sum_{\mathrm{angle}/\mathrm{angle}}k_{aa}(\theta -\theta_{0})(\theta^{\prime }-{\theta^{\prime }}_{0})+\\ +\sum_{\mathrm{bond}/\mathrm{torsion}}(b-b_{0})[k_{bt1}\cos\phi +k_{bt2}\cos2\phi +k_{bt3}\cos3\phi ]+\sum_{\mathrm{angle}/\mathrm{torsion}}(\theta -\theta_{0})[k_{at1}\cos\phi +k_{at2}\cos2\phi +k_{at3}\cos3\phi ]\\ +\sum_{\mathrm{angle}/\mathrm{torsion}/\mathrm{angle}}k_{ata}(\theta -\theta_{0})(\theta^{\prime }-{\theta^{\prime }}_{0})\cos\phi +\sum_{nonbond}\varepsilon_{ij}\left[2{\left(\frac{r_{ij0}}{r_{ij}}\right)}^{9}-3{\left(\frac{r_{ij0}}{r_{ij}}\right)}^{6}\right]+\sum_{\mathrm{nonbond}}\frac{q_{i}q_{j}}{4\pi \varepsilon_{0}r_{ij}} \end{array}$$

In the last equation, the first four sums denote the energies required to stretch bonds (*b*), bend angles (*θ*), change torsion angles (*ϕ*) by twisting atoms about the bond axis, and distort atoms out of the plane (*χ*) formed by the atoms to which they are bonded. The next six sums denote the energies between the four types of internal coordinates described as functions of the Cartesian atomic coordinates [31]. The final two sums that contain functions of the atom pair distance *q_ij_* denote the Lennard–Jones-based van der Waals (vdW) non-bond interactions and the Coulomb’s electrostatic non-bond interactions due to the charges *q_i_* and *q_j_*, respectively. The subscript 0 found in some parameters denotes corresponding reference values. The constant *ε*_0_ is the well-known vacuum permittivity. Depending on the atom-type combinations, the COMPASS force field predefines the stiffness-like parameters *k_b_*_2_, *k_b_*_3_, *k_b_*_4_, *k_a_*_2_, *k_a_*_3_, *k_a_*_4_, *k_t_*_1_, *k_t_*_2_, *k_t_*_3_, *k_χ_*, *k_bb_*, *k_ba_*, *k_aa_*, *k_bt_*_1_, *k_bt_*_2_, *k_bt_*_3_, *k_at_*_1_, *k_at_*_2_, *k_at_*_3_, and *k_ata_* as well as the functional form of each term *q_i_*, *q_j_*, *ε_ij_*, and *r_ij_*_0_. Here, the vdW contributions are computed according to the atom-based summation method using a cut-off radius of 12.5 Å and long-range corrections, while the electrostatic contributions are computed by adopting the Ewald summation method with an accuracy of 0.001 kcal/mol [34].

Evidently, the relevant positioning of the molecules is performed after computing the interactions between neighbor atoms via the COMPASS force field whereas the single chain conformations, ring spearing, and close contacts are constantly monitored. To achieve a minimized initial unit cell state, low-energy sites are preferred over high-energy sites for each molecular structure. A GO process, as the one described earlier, is executed to additionally reduce the overall potential energy of the 3D problem domain.

### 3.3. NPT Dynamic Analysis of the Unit Cells

All the MD simulations take place under the NPT ensemble and by using a time step of 1 fs. The external pressure of the unit cell is maintained at 1 atm throughout each dynamic analysis. After the finalization of the procedure at a specific temperature level, the relaxed equilibrium state, true final density, and side lengths of the unit cell are obtained. Performing a dynamic analysis by introducing additional time intervals under different ensembles such as NVT or using a time step lower than 1 fs has no observable effect on the final numerical solutions. Due to the dynamic nature of the simulation, in order to keep the system under a specific temperature and pressure level, the Andersen thermostat and Berendsen barostat are utilized, respectively [34].

### 3.4. Thermomechanical Properties Calculation

After achieving equilibrium at a given temperature *T*, the elastic properties are computed by applying to the 3D unit cells a set of three pairs of uniaxial tension/compression and three pairs of pure shear static strains of a maximum amplitude of ±0.001.

The stresses at each strain level may be estimated through the following average virial stress of a system of particles [34]:

(5) $$\sigma_{\mathrm{av}}=\frac{1}{2V}\sum_{j(\neq i)}r_{ij}⊗f_{ij}$$

where *V* is the volume of the system, *i* and *j* denote two particles at positions **r***_i_* and **r***_j_*, respectively, **r***_ij_* is equal to **r***_j_* − **r***_i_*, and **f***_ij_* is the inter-particle force applied on particle *i* by particle *j*.

By considering the symmetry of the stress, strain, and stiffness tensors, Hooke’s law may be expressed as:

(6) σ=Cε

Since the nanocomposite is assumed to be isotropic, the Lamé coefficients *λ* and *μ* may be defined by diagonal stiffness coefficients of **C** as:

(7) $$\lambda =\frac{1}{3}(C_{11}+C_{22}+C_{33})-\frac{2}{3}(C_{44}+C_{55}+C_{66})$$

(8) $$\mu =\frac{1}{3}(C_{44}+C_{55}+C_{66})=G$$

where *G* is the shear modulus.

Evidently, the elastic modulus *E* and the Poisson’s ratio *ν* may be calculated, respectively, by the following equations:

(9) $$E=\frac{\mu (3\lambda +2\mu )}{\lambda +\mu }$$

(10) $$\nu =\frac{\lambda }{2(\lambda +\mu )}$$

The computation of the initial and final unit cell volume *V*_0_ and *V*_1_, respectively, at a reference temperature *T*_0_ and an arbitrary temperature *T*_1_ > *T*_0_, respectively, enables the estimation of the volume coefficient of thermal expansion *a_V_* via the equation:

(11) $$a_{V}(T_{0}\leq T\leq T_{1})=\frac{V_{1}-V_{0}}{T_{1}-T_{0}}\frac{1}{V_{0}}$$

Finally, the linear coefficient of thermal expansion *a_L_* for an isotropic medium may be approximated by:

(12) $$a_{L}=a_{V}/3$$

## 4. Second Simulation Scale: FEM Formulation

### 4.1. Geometry Definition and Finite Element Discretization

**Figure 4:**
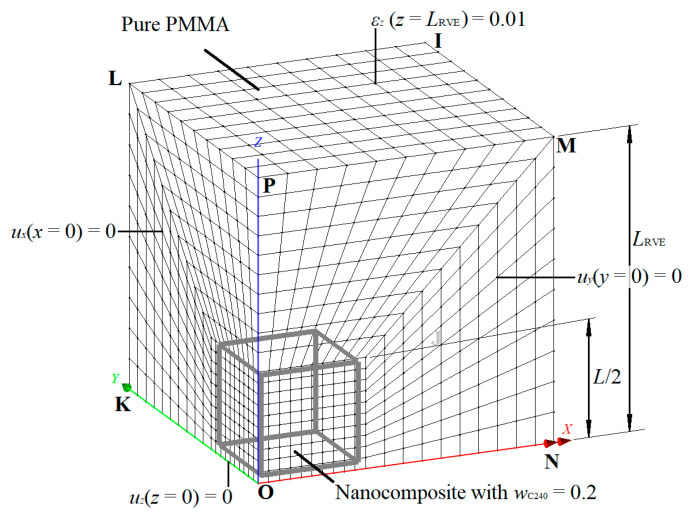
Geometry, boundary conditions, and finite element discretization of the nanocomposite RVE with a mass fraction of *w*_C240_ = 0.05 (1/8 of the periodic volume of the whole system domain).

At the second scale, nanocomposites with small mass fractions of C_240_ are modeled and simulated through FEM. A representative FEM model of the RVE, which corresponds to the case *w*_C240_ = 0.05, is illustrated in Figure 4 and defined by the IJKLMNOP cubic domain. The problem is analyzed according to a global Cartesian coordinate system (*x*, *y*, *z*) positioned at the vertex O. As depicted in the figure, the RVE is consisted of the C_240_/PMMA subdomain with *w*_C240_ = 0.2 and the outer pure PMMA subdomain.

The edge length of the nanocomposite subdomain with *w*_C240_ = 0.2 is taken equal to *L*/2, where *L* is the corresponding unit cell length computed via the MD-only simulation at the first scale analysis. On the other hand, Equations (1) to (2) may be combined in order to estimate the length of the RVE *L*_RVE_ as follows:

(13) $$L_{\mathrm{RVE}}=\frac{1}{2}\sqrt[{3}]{\frac{m_{C240}}{w_{C240}\rho_{C240}}}$$

**Figure 5:**
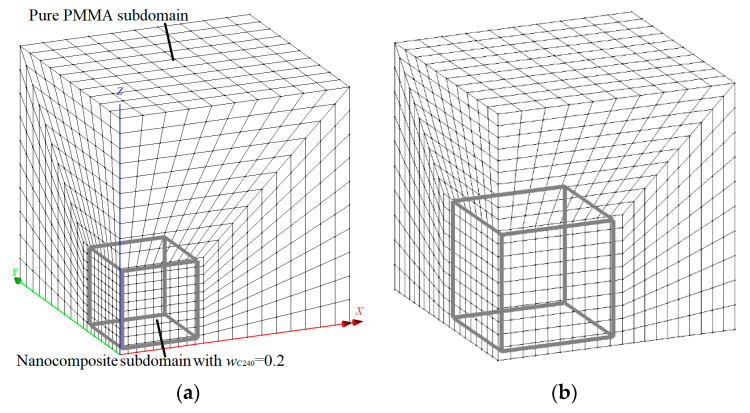

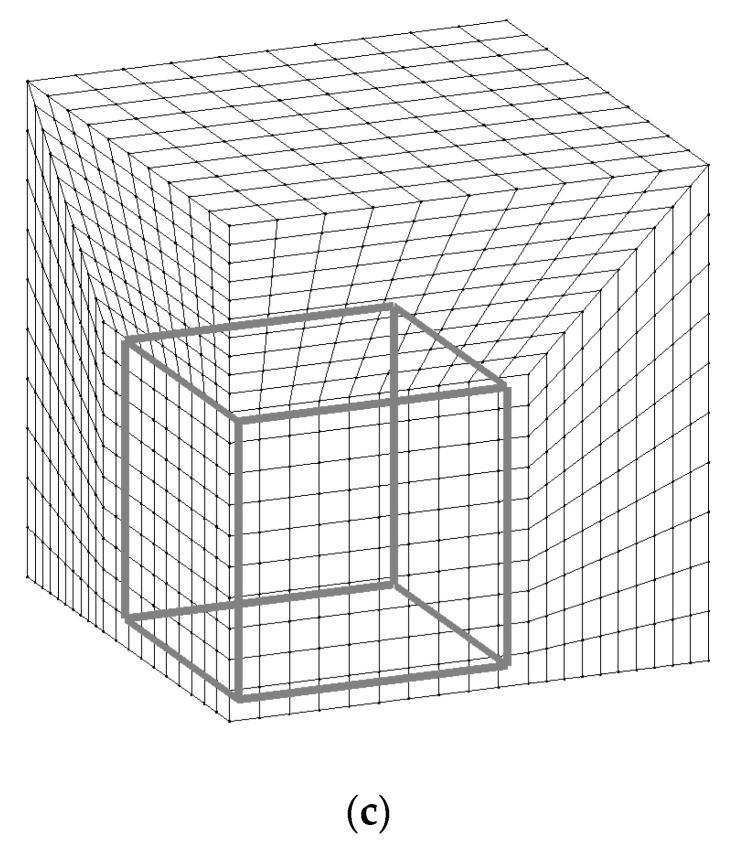
Finite element discretization of the nanocomposite RVE with a mass fraction of (**a**) *w*_C240_ = 0.01, (**b**) *w*_C240_ = 0.03, and (**c**) *w*_C240_ = 0.05 (1/8 of the periodic volume of the whole system domain).

Both subdomains are discretized with isoparametric, hexahedral, linear, eight-noded finite elements that have four degrees of freedom per node, i.e., the displacements *u_x_*, *u_y_*, *u_z_*, and the temperature *T* [33]. The finite element meshes for the three case studies *w*_C240_ = 0.01, 0.03, and 0.05 are depicted in Figure 5a–c, respectively.

### 4.2. Material Properties Input and Output

The properties of both subdomains of each RVE FEM model are considered as temperature-dependent elastic. The elastic modulus and Poisson’s ratio of both the nanocomposite with *w*_C240_ = 0.2 and the pure PMMA are input as functions of temperature, i.e., *E*(*T*) and *ν*(*T*). These functions are determined by fitting corresponding data points computed by the MD-only simulations at the first scale of the analysis. In order to compute the elastic properties of the nanocomposite RVE, appropriate boundary conditions are applied. For the calculation of the elastic modulus *E*_RVE_(*T*) and the Poisson’s ratio *ν*_RVE_(*T*), a uniform strain of *ε_z_*(*T*) = 0.001 is applied on the edge *z* = *L*_RVE_. Simultaneously, the constraints *u_x_* = 0, *u_y_* = 0, and *u_z_* = 0 are applied on the edges *x* = 0, *y* = 0, and *z* = 0, respectively, while the edges *x* = *L*_RVE_ and *y* = *L*_RVE_ are kept parallel to their original shape by nodal coupling, since the symmetry implies that shear stresses on these edges should be zero. Then, the elastic modulus of the nanocomposite *E*_RVE_(*T*) is calculated from the ratio of average stress *σ*_zav_(*T*), which are obtained from the sum of reactions in the ground edge *z* = 0 to the applied strain *ε_z_*(*T*) = 0.001. Finally, the Poisson’s ratio *ν*_RVE_(*T*) is estimated by the ratio of the arisen average transverse strain *ε_x_*_av_(*T*) = *ε_y_*_av_(*T*) to the applied normal strain *ε_z_*(*T*) = 0.001.

Considering the FEM simulation of the RVE thermal expansion behavior, a different load case is applied. Evidently, the constraints remain the same in accordance with the symmetry. The linear coefficient of thermal expansion *a_L_*(*Τ*) of both subdomains is defined according to the corresponding output from the MD-only simulations again. Then, a small temperature load increment Δ*T* = *T*_1_−*T*_0_ is applied in the whole RVE to compute its arisen edge length increment Δ*L*_RVE_ = *L*_RVE1_ − *L*_RVE0_. As a result, the linear coefficient of thermal expansion of the RVE may be estimated by the following relationship:

(14) $$a_{L_{\mathrm{RVE}}}(T_{0}\leq T\leq T_{1})=\frac{L_{\mathrm{RVE}1}-L_{\mathrm{RVE}0}}{T_{1}-T_{0}}\frac{1}{L_{\mathrm{RVE}0}}$$

## 5. Results and Discussion

### 5.1. First Simulation Scale

**Figure 6:**
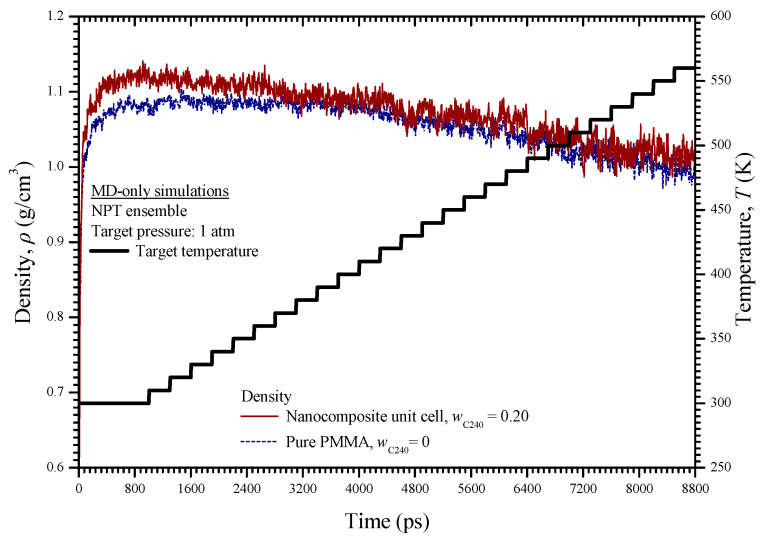
Density of the pure PMMA and nanocomposite with *w*_C240_ = 0.2 versus time in contrast with the time-dependent temperature during the MD simulations.

For both material cases under investigation, i.e., the pure PMMA and the PMMA reinforced with fullerene C_240_ at a mass fraction of 0.2, the MD simulations are conducted under the NPT ensemble with a target pressure of 1 atm and a time-dependent temperature *T*, which varies from 300 to 560 K as defined in Figure 6. For the figure, it may be seen that initially, the target temperature is considered stable at 300 K for a time interval of 1000 ps, so that both the pure PMMA unit cell and the nanocomposite unit cell with *w*_C240_ = 0.2 reach a minimized energy and an equilibrium state. Then, it is assumed that temperature exhibits a step increment with time. Specifically, at each step, the temperature remains stable for 300 ps and then increases by 10 K. Following such a technique, the equilibrium is achieved much faster at each temperature level, expect for the first investigated temperature level at 300 K for which a longer time interval is required for convergence.

**Figure 7:**
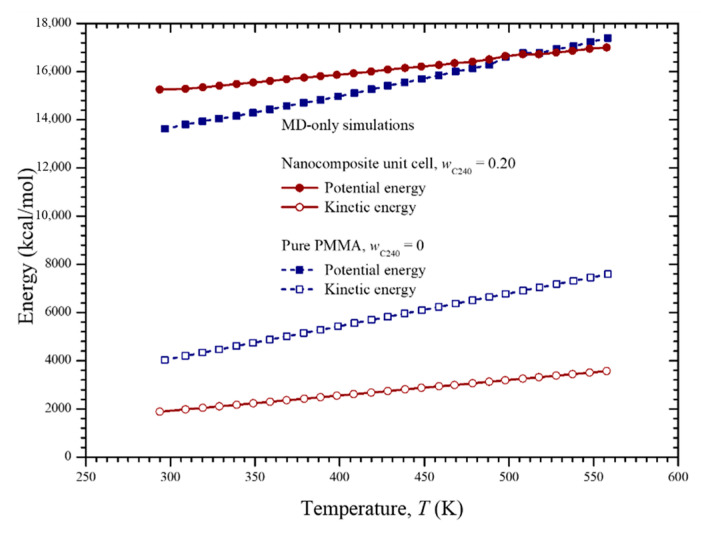
Potential and kinetic energy of the pure PMMA and nanocomposite with *w*_C240_ = 0.2 with respect to the temperature.

The density variation of the pure PMMA and the C_240_/PMMA nanocomposite unit cells during the dynamic analysis may be seen in Figure 6. Note that the initial density of 0.6 g/cm^3^ assumed for both unit cells increases to reach its proper value at room temperature and then decreases as the temperature elevates. The variation of the potential and kinetic energies of the two cells with temperature is illustrated in Figure 7. All variations are almost linear ascending, while the kinetic energy of the pure PMMA is higher for the whole temperature range and presents a higher gradient in comparison with the nanocomposite unit cell. On the other hand, the pure PMMA presents a lower potential energy up to 500 K in contrast with its reinforced version with *w*_C240_ = 0.2.

**Figure 8:**
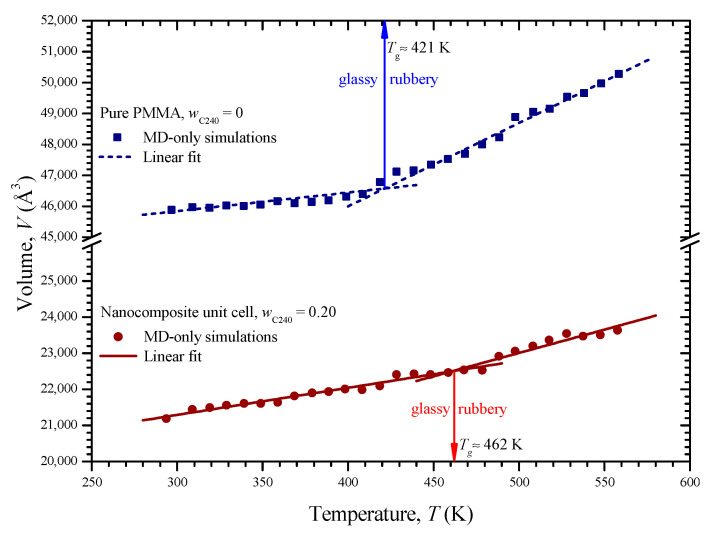
Volume of the pure PMMA and nanocomposite with *w*_C240_ = 0.2 with respect to temperature and the corresponding glass transition temperatures *T*_g_ arisen from the intersection of appropriate linear fittings.

Figure 8 depicts the volume change of the two unit cells versus temperature. As it can be seen, the volume variation of each unit cell is characterized by two different regions, i.e., the glassy and the rubbery one. At each region, the MD data points imply a linear behavior of a different slope. A linear regression is applied on the set of data of the glassy and rubbery region of each medium. Then, the glass transition temperatures *T*_g_ are estimated from the intersection of the arisen lines at 421 and 462 K for the pure and reinforced PMMA, respectively. The glass transition temperature is considerably increased by reinforcing the PMME with fullerene C_240_ at a weight concentration of 20%. A similar phenomenon has been observed regarding the case of PMMA filled with functionalized graphene [10]. The computed *T*_g_ regarding the pure PMMA is in good agreement with the corresponding values 411.4 and 430 K, which have been predicted in the MD-based studies [10] and [29], respectively. The details about all the linear regressions shown in Figure 8 may be found in Table 1. Using the fitting parameters of the table, one may define the volume of the unit cells as a function of temperature.

**Figure 9:**
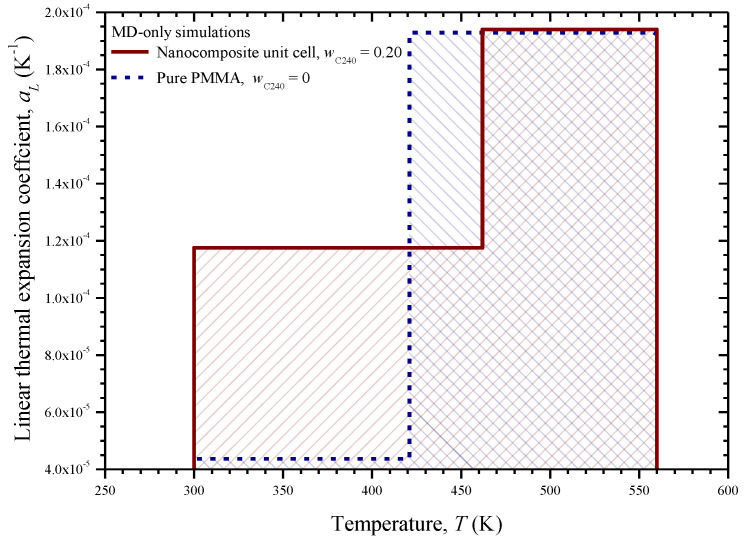
Linear thermal expansion coefficient of the pure PMMA and the nanocomposite with *w*_C240_ = 0.2 unit cell with respect to the temperature.

The linear coefficient of thermal expansion *a_L_* of the pure PMMA and the nanocomposite with *w*_C240_ = 0.2 with respect to the temperature is illustrated in Figure 9. The relevant estimations are based on Equations (11) and (12). As observed for both materials, the linear coefficient of thermal expansion has a lower and constant value for the temperatures below *T*_g_, while it exhibits a notable constant increase after this temperature point and up to 560 K. The presence of C_240_ within the PMMA matrix, as expected [10], appears to lead to a rise in thermal expansion, especially for temperatures below 421 K, i.e., the glass transition point of the matrix.

The calculated *a_L_* of the pure PMMA is 4.4 × 10^−5^ and 1.9 × 10^−4^ K^−1^ in the glassy and rubbery state, respectively, which are in decent agreement with the corresponding values 7.3 × 10^−5^ K^−1^ and 2.6 × 10^−4^ K^−1^, as reported in a different MD analysis [10]. Note that experimental evidence [35] suggests that the pure PMMA presents a coefficient of linear thermal expansion in the range from 5 × 10^−5^ to 9 × 10^−5^ K^−1^ at room temperature (glassy state).

**Figure 10:**
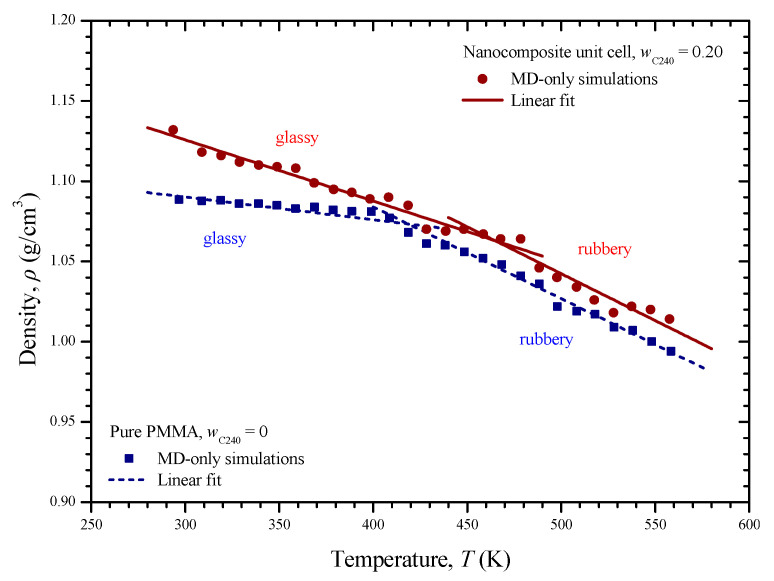
Density of the pure PMMA and nanocomposite with *w*_C240_ = 0.2 with respect to the temperature and corresponding linear fittings.

The dependence of the density *ρ* on the temperature *T* is illustrated for the pure PMMA and nanocomposite unit cell in Figure 10. The density for both cases follows a linear decrease characterized by two slopes. The kink positions reveal the corresponding glass transition points, which are identical with those found from Figure 8. At all temperature levels, the density of the nanocomposite remains higher. Again, a two-slope linear regression of the density–temperature variations is performed, the results of which may be found in Table 1.

**Figure 11:**
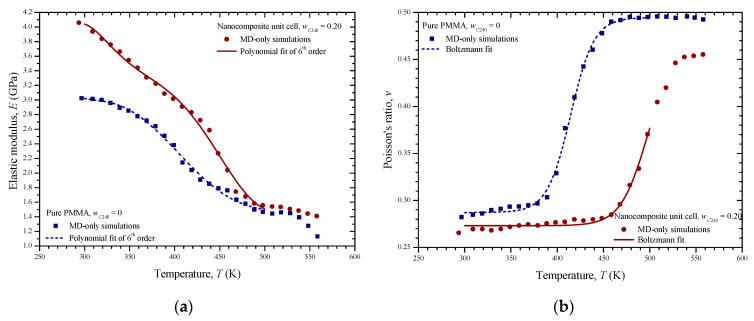
The (**a**) elastic modulus and (**b**) Poisson ratio of the pure PMMA and the nanocomposite with *w*_C240_ = 0.2 with respect to the temperature and corresponding nonlinear fittings up to 500 K.

The elastic modulus of the two unit cells with respect to the temperature is shown in Figure 11a. It becomes obvious that the C_240_/PMMA nanocomposite presents an advanced stiffness. This is due to the enhanced stiffness of the carbon nanoparticle. As expected, a stress relaxation is observed at the glass transition points for both materials, which is implied by the significant drop of elastic modulus. The elastic modulus–temperature nonlinear variations are fitted well with polynomial functions of 6th degree that are fully defined in Table 1 for temperatures up to 500 K.

The temperature dependence of Poisson’s ratio of the PMMA and nanocomposite is illustrated in Figure 11b. The Poisson ratio tends to reach the value of 0.5 as the temperature increases for the pure PMMA case. On the other hand, the Poisson ratio of the PMMA reinforced with C_240_ at a mass fraction of 20% presents lower values due to the effects of the fullerene constituent. An obvious intense Poisson ratio increase occurs nearby the *T*_g_ point. Table 1 includes details about the fitting of the two Poisson ratio–temperature variations for the temperature range from 300 to 500 K with the Boltzmann sigmoid function, which are defined in the table as well.

In order to evaluate the performance of the MD-only simulations, Table 2 is presented. The table includes some comparisons between the present results regarding the pure PMMA mechanical properties at the room temperature with other corresponding predictions.

### 5.2. Second Simulation Scale

Three small C_240_ loadings of 1%, 3%, and 5% by weight are investigated by using the FEM models shown in Figure 5a–c. The linear coefficient of thermal expansion, elastic modulus, and Poisson’s ratio of the pure PMMA subdomain and nanocomposite subdomain with a C_240_ concentration of 20 wt % are inserted into the model by utilizing the temperature-dependent functions provided from the first-scale MD analysis and illustrated in Figure 11a,b and Figure 9, respectively. The same number of finite elements is used in all cases. Denser meshes than the ones depicted in Figure 5 lead to negligibly different numerical solutions.

**Figure 12:**
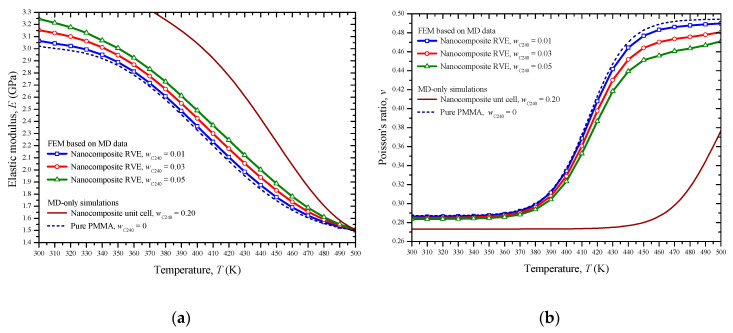
The (**a**) elastic modulus and (**b**) Poisson ratio of the nanocomposite with small fullerene mass fractions with respect to the temperature, predicted by combining FEM and MD.

Figure 12a,b presents the elastic modulus *E* and the Poisson ratio *ν* of the tested C_240_/PMMA nanocomposites, respectively. The limit cases for *w*_C240_ = 0 and *w*_C240_ = 0.2 treated with the MD-only method are included in these figures for comparison reasons. A nonlinear reduction of the mechanical performance, as expressed by the elastic modulus decrease and Poisson ratio increase, is observed for all the materials as the temperature rises. Contrary, the higher the fullerene mass fraction, the higher the elastic modulus and the lower the Poisson ratio. For a given temperature, almost a linear change occurs in these properties as the reinforcement concentration increases.

Table 3 shows a qualitative comparison between some present estimations via the proposed multiscale analysis and others predicted via MD [15] in which a rather dissimilar fullerene structure such as the carbon onion C_60_@C_240_ has been considered. Unfortunately, to the author’s best knowledge, there is not any relevant experimental contribution regarding the stiffness of the C_240_/PMMA nanocomposite, in order to provide a more comprehensive assessment. However, regarding the elastic modulus of the pure PMMA, a comparison with a corresponding experimental value is included in Table 2.

**Figure 13:**
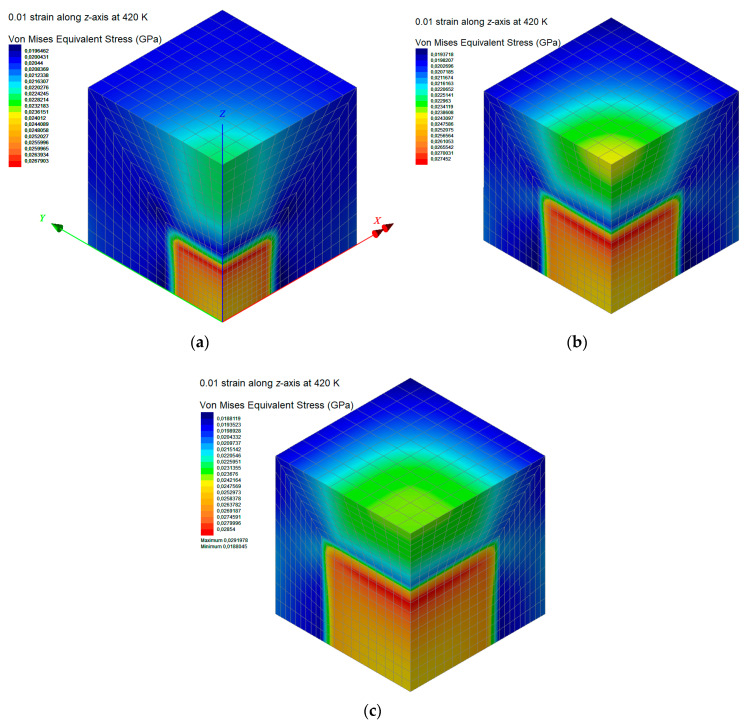
Contours of the resultant von Mises equivalent stress of the nanocomposite RVE at a temperature of 400 K, with mass fractions of (**a**) *w*_C240_ = 0.01, (**b**) *w*_C240_ = 0.03, and (**c**) *w*_C240_ = 0.05.

The contours of the von Mises equivalent stress of the nanocomposite with *w*_C240_ = 0.01, 0.03, 0.05 and for a temperature of 420 K are depicted in Figure 13a–c. As seen, the maximum equivalent stress, which is located in the nanocomposite subdomain with *w*_C240_ = 0.2, rises as the fullerene concentration is increased. This reveals an enhanced capability of the fullerene to carry loads.

**Figure 14:**
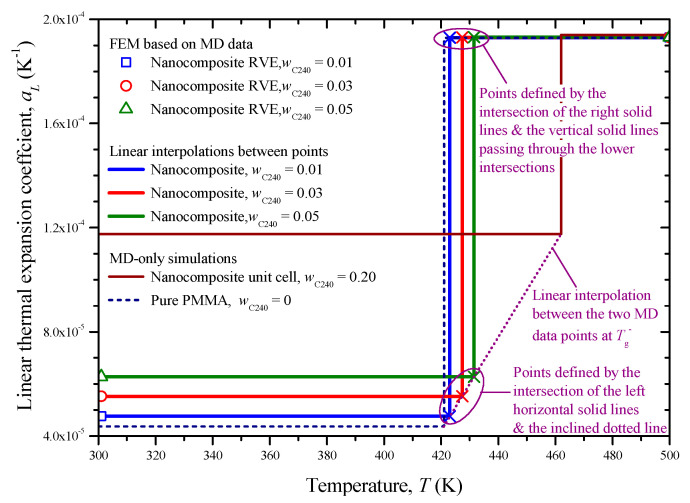
Linear thermal expansion coefficient of the nanocomposite with small fullerene mass fractions with respect to the temperature, predicted by combining FEM and MD as well as using linear interpolations.

The proposed FEM analysis is not efficient enough to compute straightforwardly the linear coefficient of thermal expansion *a_L_* for the whole temperature range from 300 to 500 K, since complex molecular phenomena occur in the interphase zone near the phase transition temperature, which may only be described via atomistic models. Thus, in order to assure that the FEM computations take place exclusively in the glassy or rubbery state of the nanocomposite with *w*_C240_ = 0.01, 0.03, and 0.05, a targeted temperature change is applied from 300 to 301 K or from 499 to 500 K, respectively. Accordingly, the two boundary values of *a_L_* in the temperature interval [300 K, 500 K] are obtained through Equation (14). These two FEM data points are inserted in Figure 14, which also includes the step functions *a_L_*(*T*) for the limit cases, investigated via MD only, where *w*_C240_ = 0 and *w*_C240_ = 0.2. To assure safe estimations for the cases *w*_C240_ = 0.01, 0.03, and 0.05, a linear interpolation is required, which is graphically realized by interconnecting the two lower corner points (*T*_g_, *a_L_*(*T*_g^−^_)) of the two step functions *a_L_*(*T*) defined by MD. Then, some good approximations of the *a_L_* of the nanocomposites with *w*_C240_ = 0.01, 0.03, and 0.05 around their *T*_g_ may be graphically derived by defining specific intersection points and making linear interpolations as Figure 14 describes in detail.

**Figure 15:**
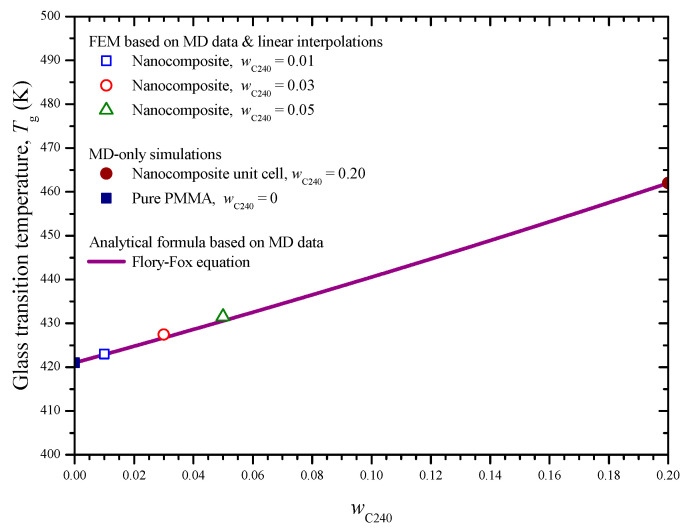
Glass transition temperature of the nanocomposite for small fullerene mass fractions, predicted by combining FEM and MD as well as using linear interpolations.

Note that the *T*_g_ points for the small fullerene concentrations are also indirectly revealed by the arisen intersection points. The good performance of the proposed graphical procedure, which is grounded on the utilization of both FEM and MD data points, in predicting *T*_g_ is demonstrated in Figure 15, where a theoretical estimation via the well known Flory–Fox equation [36] is included. The specific equation is defined as follows:

(15) $$\frac{1}{T_{g}(w_{C240})}=\frac{w_{C240}}{T_{g}{}_{C240}}+\frac{1-w_{C240}}{T_{g}{}_{\mathrm{PMMA}}}$$

where *T*_gC240_ are *T*_gPMMA_ is the glass transition temperature of the component C_240_ and pure PMMA, respectively, while the nanocomposite *T*_g_(*w*_C240_) function is assumed to pass through the two MD data points.

**Figure 16:**
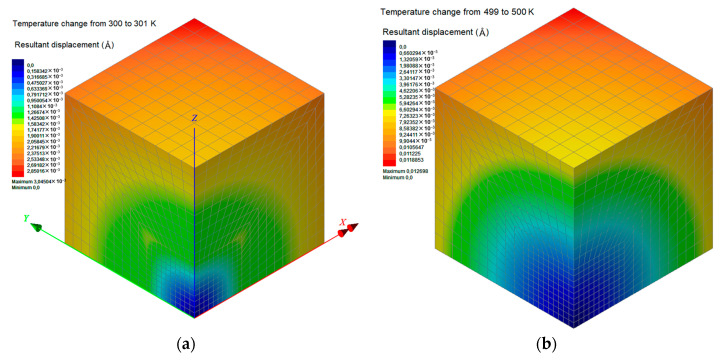
Contours of the resultant displacement of the nanocomposite RVE with a mass fraction with *w*_C240_ = 0.01, for a temperature change (**a**) from 300 to 301 K and (**b**) from 499 to 500 K.

**Figure 17:**
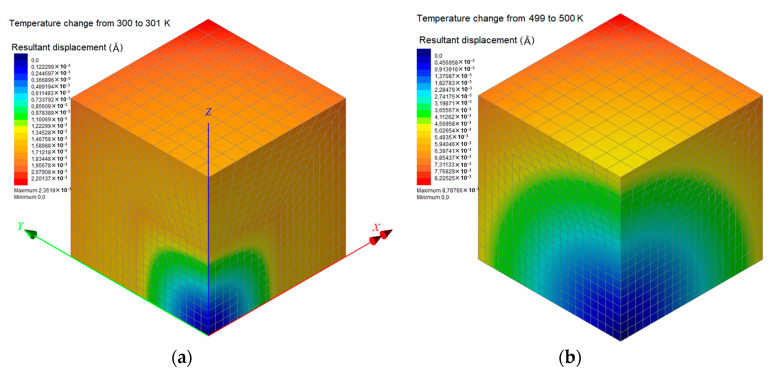
Contours of the resultant displacement of the nanocomposite RVE with a mass fraction with *w*_C240_ = 0.03, for a temperature change (**a**) from 300 to 301 K and (**b**) from 499 to 500 K.

**Figure 18:**
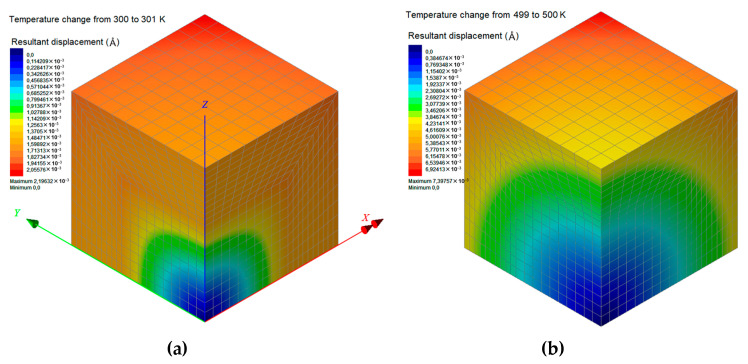
Contours of the resultant displacement of the nanocomposite RVE with a mass fraction with *w*_C240_ = 0.05, for a temperature change (**a**) from 300 to 301 K and (**b**) from 499 to 500 K.

Figure 16a,b present the colored distributions of the resultant displacement due to a temperature change from 300 to 301 K (glassy state) and from 499 to 500 K (rubbery state), respectively, for the nanocomposite with *w*_C240_ = 0.01. It becomes obvious that the consequent expansions are more intense at a higher temperature level since the coefficients of thermal expansion for all subdomains are higher in their glassy state. Similar contours are presented in Figure 17 and Figure 18 for the cases *w*_C240_ = 0.03 and 0.05, respectively, leading to analogous conclusions.

## 6. Conclusions

A theoretical attempt was made to provide a multiscale numerical formulation for the efficient prediction of the thermoelastic response of nanomaterial/polymer composites. The aim was to provide an accurate numerical tool of a low computational cost that is capable of treating large problem domains, which would require substantial resources if treated via atomistic methods alone. To deal with such problems, the proposed method is applied into two phases. It starts from the molecular scale via MD and ends up to the continuum scale via FEM. Thus, the hybrid simulation is capable of capturing the complicated atomistic and interphase phenomena as well as reducing the computational effort simultaneously.

For the purpose of the study, the fullerene C_240_ and the PMMA were utilized as the reinforcement and the matrix material, respectively. A MD formulation was initially developed in order to predict the temperature-dependent elastic modulus, Poisson ratio, and linear coefficient of thermal expansion of the pure PMMA and the C_240_/PMMA with a high fullerene mass fraction. The glass transition temperature of the specific media was also defined by the change in the slope of relevant thermal expansion curves. The extracted data points were fitted via appropriate functions and inserted into several FEM models to simulate nanocomposites with smaller fullerene mass fractions. The computations showed that the proposed multiscale formulation may perform well for low nanofiller contents up to 5 wt %.

The FEM computations led to the full definition of the same properties for the whole investigated temperature and fullerene mas fraction range. It was demonstrated that for a given temperature level, the rise of the fullerene mass fraction leads to an almost linear increase of the nanocomposite stiffness but also to an analogous decrease of its Poisson’s ratio. The linear coefficient of thermal expansion of the nanocomposite was found to be constant before and after the glass transition temperature. Its value was significantly higher for the glassy state, while it showed a nearly linear increase with the increase of the nanofiller mass fraction. Finally, a drastic drop of the nanocomposite mechanical performance was observed near the glass transition point due to the stress relation.

A further investigation is planned to be made in a future work, where the effects of utilizing different fullerene sizes, several combinations of fullerene types, and non-uniform nanofiller distributions will be extensively studied.

## Figures and Tables

**Table 1:** Definition of all the fitting functions adopted throughout the analysis.

*Fitting of MD Data with Functions of Temperature*						
Property	Fitting Equation (*T*→K)	Nanocomposite with *w*_C240_ = 0.02			Pure PMMA	
Volume, *V* (Å^3^)	Linear *V* = *f* + *gT*	*T* range	300 ≤ *T* ≤ 462	462 < *T* ≤ 560	300 ≤ *T* ≤ 421	421 ≤ *T* ≤ 560
		*f*	19,038.73973	16,537.08047	44,046.02025	35,216.40195
		*g*	7.50775	13.10966	6.0055	26.96125
		*Adjusted R* ^2^	0.96339	0.89922	0.83912	0.98609
Density, *ρ* (g/cm^3^)	Linear *ρ* = *h* + *lT*	*T* range	300 ≤ *T* ≤ 462	462 < *T* ≤ 560	300 ≤ *T* ≤ 421	421 < *T* ≤ 560
		*h*	1.2401	1.33445	1.13239	1.3125
		*l*	−3.81086 × 10^−4^	−5.84103 × 10^−4^	−1.40928 × 10^−4^	−5.71409 × 10^−4^
		*Adjusted R* ^2^	0.96111	0.91912	0.85399	0.98549
Elastic modulus, *E* (GPa)	6th order polynomial *E* = *A* + *B*_1_*T* + *B*_2_*T*^2^ + *B*_3_*T*^3^ + *B*_4_*T*^4^ + *B*_5_*T*^5^ + *B*_6_*T*^6^	*T* range	300 ≤ *T* ≤ 500		300 ≤ *T* ≤ 500	
		*A*	−2209.3885		409.32795	
		*B*_1_	33.54518		−5.76718	
		*B*_2_	−0.20948		0.0332	
		*B*_3_	6.90037 × 10^−4^		−9.88824 × 10^−5^	
		*B*_4_	−1.26453 × 10^−6^		1.60037 × 10^−7^	
		*B*_5_	1.22205 × 10^−9^		−1.33008 × 10^−10^	
		*B*_6_	−4.86522 × 10^−13^		4.40984 × 10^−14^	
		*Adjusted R* ^2^	0.99509		0.99509	
Poisson’ s ratio, *ν*	Boltzmann *ν* = (*D*_1_ − *D*_2_)/{1 + exp[(*T* − *τ*_0_)/*τ*]} + *D*_2_	*T* range	300 ≤ *T* ≤ 500		300 ≤ *T* ≤ 500	
		*D*_1_	0.2731		0.28715	
		*D*_2_	0.46094		0.49459	
		*τ*_0_	497.04191		414.53197	
		*τ*	14.45546		12.68674	
		*Adjusted R* ^2^	0.9982		0.99698	

**Table 2:** Comparison of the elastic properties of the pure PMMA computed here via MD, with corresponding results from other studies.

Study	Materials Properties of Pure PMMA at *T* = 300 K	
	Elastic Modulus, *E* (GPa)	Poisson’s Ratio, *ν*
Present MD-only formulation	3.065	0.286
Different MD formulation [15]	3.052	0.257
Experimental [26]	3.400	-

**Table 3:** Present elastic properties of the nanocomposite with *w*_C240_ = 0.05 in contrast with some comparable results from another theoretical study.

Theoretical Study	Material Properties at *T* = 300 K	
	Elastic Modulus, *E* (GPa)	Poisson’s Ratio, *ν*
Present FEM combined with MD of PMMA reinforced with C_240_ at 5.00 wt %	3.247	0.284
Different MD simulation [15] of PMMA reinforced with onion C_60_@C_240_ at 5.01 wt %	3.590	0.271
